# Value of Imaging in the Non-Invasive Prediction of Recurrence after Catheter Ablation in Patients with Atrial Fibrillation: An Up-to-Date Review

**DOI:** 10.31083/j.rcm2408241

**Published:** 2023-08-18

**Authors:** Mengyuan Jing, Dong Li, Huaze Xi, Yuting Zhang, Junlin Zhou

**Affiliations:** ^1^Department of Radiology, Lanzhou University Second Hospital, 730030 Lanzhou, Gansu, China; ^2^Second Clinical School, Lanzhou University, 730030 Lanzhou, Gansu, China; ^3^Key Laboratory of Medical Imaging of Gansu Province, 730030 Lanzhou, Gansu, China; ^4^Gansu International Scientific and Technological Cooperation Base of Medical Imaging Artificial Intelligence, 730030 Lanzhou, Gansu, China; ^5^Department of Cardiovascular Medicine, Lanzhou University Second Hospital, 730030 Lanzhou, Gansu, China

**Keywords:** atrial fibrillation, catheter ablation, echocardiography, computed tomography, magnetic resonance imaging, machine learning

## Abstract

Catheter ablation (CA) is the first-line treatment for atrial fibrillation (AF) patients. However, the risk of recurrence associated with CA treatment should not be ignored. Therefore, the preoperative identification of patients at risk of recurrence is essential for identifying patients who will benefit from non-invasive surgery. Echocardiography, computed tomography (CT), and magnetic resonance imaging (MRI) are essential for the preoperative non-invasive prediction of AF recurrence after CA. Compared to laboratory examinations and other examination methods, these modalities can identify structural changes in the heart and assess functional variations. Accordingly, in past studies, morphological features, quantitative parameters, and imaging information of the heart, as assessed by echocardiography, CT, and MRI, have been used to predict AF recurrence after CA noninvasively. This review summarizes and discusses the current research on echocardiography, CT, MRI, and machine learning for predicting AF recurrence following CA. Recommendations for future research are also presented.

## 1. Introduction

Atrial fibrillation (AF) is the most common type of sustained arrhythmia in clinical settings, with prevalence and incidence increasing over the years and posing serious survival and economic burdens [1, 2]. Catheter ablation (CA) and antiarrhythmic drugs are the primary modalities to treat AF patients [3]. With advances in the technology of CA, it has become significantly superior to antiarrhythmic drugs in maintaining sinus rhythm in patients with persistent AF and has become an effective treatment modality for patients with drug-refractory AF [4, 5]. In addition, CA has been outperformed by antiarrhythmic drugs in reducing cardiovascular death and all-cause mortality in patients with AF [6]. Therefore, CA is being increasingly recommended as first-line therapy for patients with AF.

However, it should be noted that some patients with AF have a recurrence rate of up to 30% following CA and that approximately 10% of cases of AF are not terminated following CA [7]. Moreover, complications caused by CA, such as atrioesophageal fistula and cardiac tamponade, should not be ignored [8]. Consequently, identifying patient groups at a higher risk of recurrent AF can help ensure a higher success rate after CA and avoid unnecessary risks associated with invasive procedures [2, 7]. This also helps clinicians develop appropriate treatment and prevention strategies for patients with AF and adjust the rhythm control approach after CA promptly [9].

It is well known that age, sex, hypertension, and AF type, as well as other clinical information, are high-risk factors for recurrence after CA [10, 11]. Moreover, biomarkers such as B-type natriuretic peptides and high-sensitivity C-reactive proteins are also associated with AF recurrence [12]. Notably, with advances in imaging technology, non-invasive imaging with high temporal and spatial resolution plays an increasingly important role and has a wide range of applications in assessing postoperative recurrence in patients with AF who undergo CA [13, 14, 15]. Accordingly, we herein review the current status of echocardiography, computed tomography (CT), and magnetic resonance imaging (MRI) in the non-invasive assessment of AF recurrence after CA. Finally, the progress of machine learning (ML) in predicting AF recurrence after CA will also be reviewed.

## 2. Use of Echocardiography for the Prediction of AF Recurrence after CA

Echocardiography is the preferred modality for evaluating left atrial (LA) structure and function because it is more convenient, cost-effective, and safer than cardiac CT and MRI [7]. Multiple studies have identified that a larger LA diameter [16, 17, 18, 19] and volume [7, 15, 20, 21] measured via transthoracic echocardiography before CA are associated with a higher probability of AF recurrence. Moreover, the LA volume index, calculated by dividing LA volume by body surface area, including the maximum, mean, minimum, and Δvolume index, was relevant to AF recurrence after CA [22, 23, 24]. Studies have reported a significantly higher LA volume index in patients with AF relapse, attributing to the association between AF recurrence and poor LA structural remodeling [22, 23, 24]. A growing series of studies also claim an independent relationship between functional indices, such as left ventricular (LV) and LA ejection fraction, and the ratio of mitral inflow velocity to mitral annular tissue velocity (E/e’) and AF recurrence after CA [16, 19, 25, 26]. Of these patients, lower LV and LA ejection fractions and higher E/e’ signify higher AF recurrence, probably because AF not only promotes the structural remodeling of LA but also leads to its functional alteration [16, 19, 25, 26]. As such, a low functional index, as assessed using ultrasound, predicts a high rate of postoperative AF recurrence.

Epicardial adipose tissue (EAT) has been recognized as a specialized visceral adipose tissue with important biological activity and endocrine and inflammatory functions [27, 28]. Studies have reported that increased EAT thickness measured using ultrasound is significantly associated with AF recurrence after CA and that this association is mainly related to pro-inflammatory cytokine release in EAT [23, 29, 30]. Moreover, speckle tracking echocardiography (STE) is an advanced, non-invasive echocardiographic technique independent of the angle of sound waves and has great potential for clinical application [7]. Nielsen *et al*. [31] found that LA strain during the reservoir and contraction phases, as assessed by two-dimensional (2D) STE, is associated with an increased risk of recurrence in patients with AF treated with CA. Koca *et al*. [23] and Ma *et al*. [32] showed that decreased LA global longitudinal strain values, measured by STE, helped identify patients at high risk of AF recurrence after CA. A meta-analysis demonstrated that lower LA peak atrial longitudinal strain values, as measured by 2DSTE, were independent predictors of AF recurrence after radiofrequency ablation [33].

**Fig. 1.**
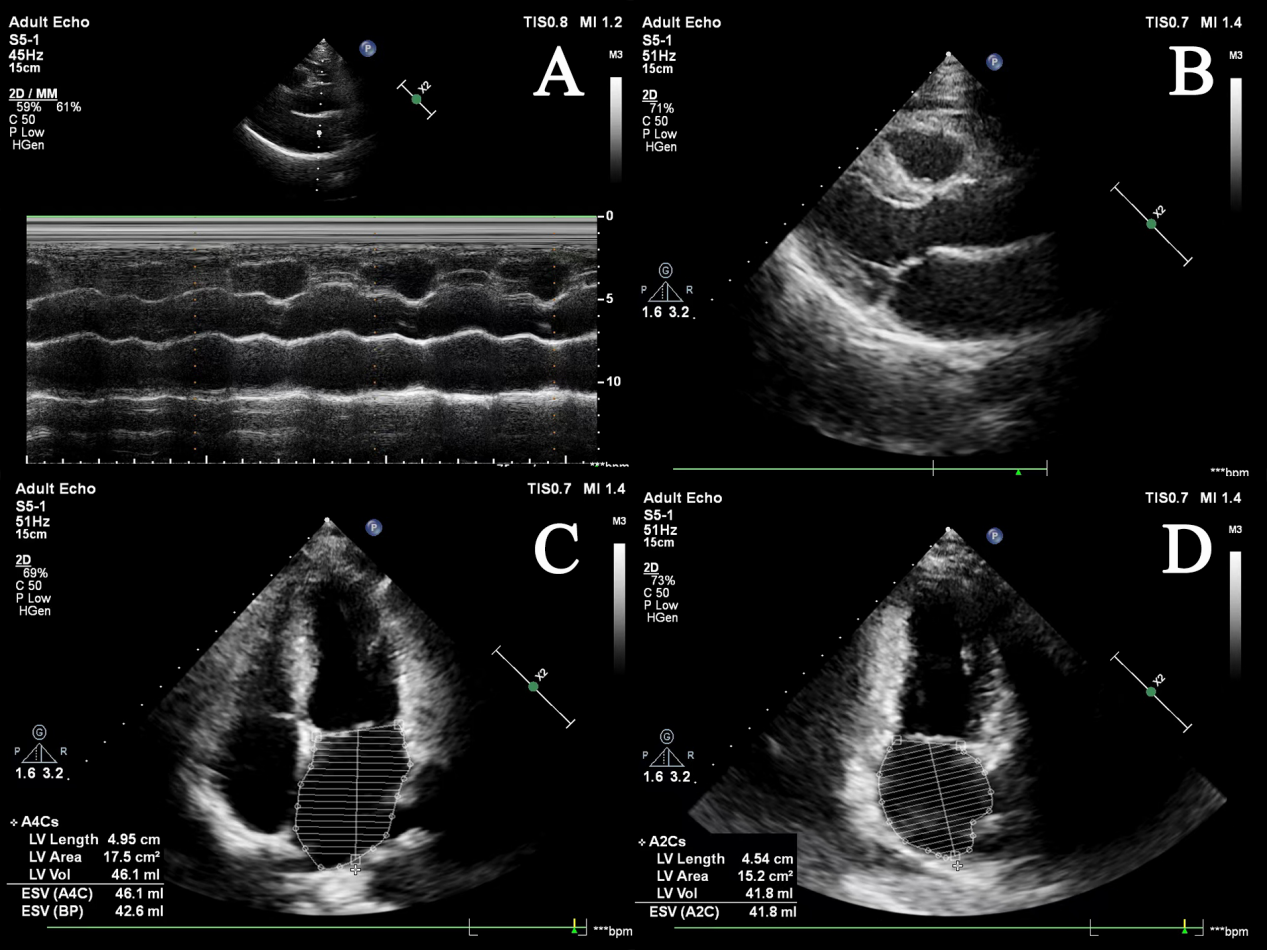
**Cardiac echocardiography images of the left atrium**. (A) M-mode of left atrial echocardiography; (B) B-mode of left atrial in long-axis view; (C) apical four-chamber view; (D) apical two-chamber view. ESV, end-systolic volume; LV, left ventricular; BP, blood pressure.

Furthermore, peak right atrial (RA) and LA longitudinal strains and their combined values were correlated with AF recurrence after CA in patients with chronic lung disease [34]. Liżewska-Springer *et al*. [35] also found that biatrial strain was the best predictor of AF recurrence after radiofrequency CA. Thus, STE is a promising tool for identifying AF recurrence following CA in clinical practice. A summary of studies using echocardiography to predict AF recurrence after CA is shown in Table 1 (Ref. [15, 16, 17, 18, 19, 20, 21, 22, 23, 24, 25, 26, 29, 30, 31, 32, 34]). Fig. 1 shows the cardiac echocardiography images.

**Table 1.** **Prediction of AF Recurrence after Catheter Ablation by Echocardiography**.

Author (reference)	Sample size	Risk factors	Follow-up time	Predictive performance
Bossard *et al*. [15]	276	Large LA volume	12 months	-
Zhao *et al*. [16]	485	LA diameter, LA ejection fraction, type of AF, and systemic inflammation score	25 ± 17 months	C-statistic = 0.741, 0.750
Ding *et al*. [17]	263	Increased neutrophil-to-lymphocyte ratio, high-sensitivity C-reactive protein and LA diameter	12 months	AUC = 0.684
Lee *et al*. [18]	263	Non-paroxysmal AF, larger LA diameter and female gender	3 years	AUC = 0.747, 0.802 and 0.789
Kim *et al*. [19]	2352	Type of AF, duration of AF, diabetes, LA diameter ≥45 mm, the ratio of mitral inflow velocity to mitral annular tissue velocity ≥10, dense spontaneous echocontrast, and decreased LAA flow velocity	5 years	AUC = 0.717
Li *et al*. [20]	87	Age, history of hypertension, LA maximum volume and LAA area change percentage	6 months	-
Lorenzo *et al*. [21]	53	Large LA volume	17 ± 2 months	-
Motoc *et al*. [22]	172	LA volume index	11.7 ± 1.6 months	AUC = 0.68
Koca *et al*. [23]	190	High LA volume index and EAT thickness, and low LA global longitudinal strain	12 ± 3 months	AUC = 0.857, 0.814 and 0.969
Soga *et al*. [24]	156	ΔLA volume index	24 months	AUC = 0.68
Shono *et al*. [25]	111	LV ejection fraction and baseline Δ LA expansion index	14.2 months	-
Chen *et al*. [26]	67	The time interval between the onset of early transmitral flow peak velocity and that of early diastolic mitral annular velocity increased	12 months	-
Canpolat *et al*. [29]	234	Thick EAT thickness	20 (IQR: 13, 24) months	AUC = 0.79
Dereli *et al*. [30]	262	Thick EAT thickness	6 months	-
Nielsen *et al*. [31]	678	LA strain during reservoir and contraction phase	12 months	-
Ma *et al*. [32]	115	LA global longitudinal strain	12 months	AUC = 0.94, 0.86
Bai *et al*. [34]	87	Peak RA and LA longitudinal strain and their combined values	32 months	C-index = 0.788, 0.759 and 0.793

AF, atrial fibrillation; LA, left atrial; AUC, area under the curve; IQR, inter quartile range; LAA, left atrial appendage; LV, left ventricle; EAT, epicardial adipose tissue; RA, right atrial.

## 3. Use of CT for the Prediction of AF Recurrence after CA

**Fig. 2.**
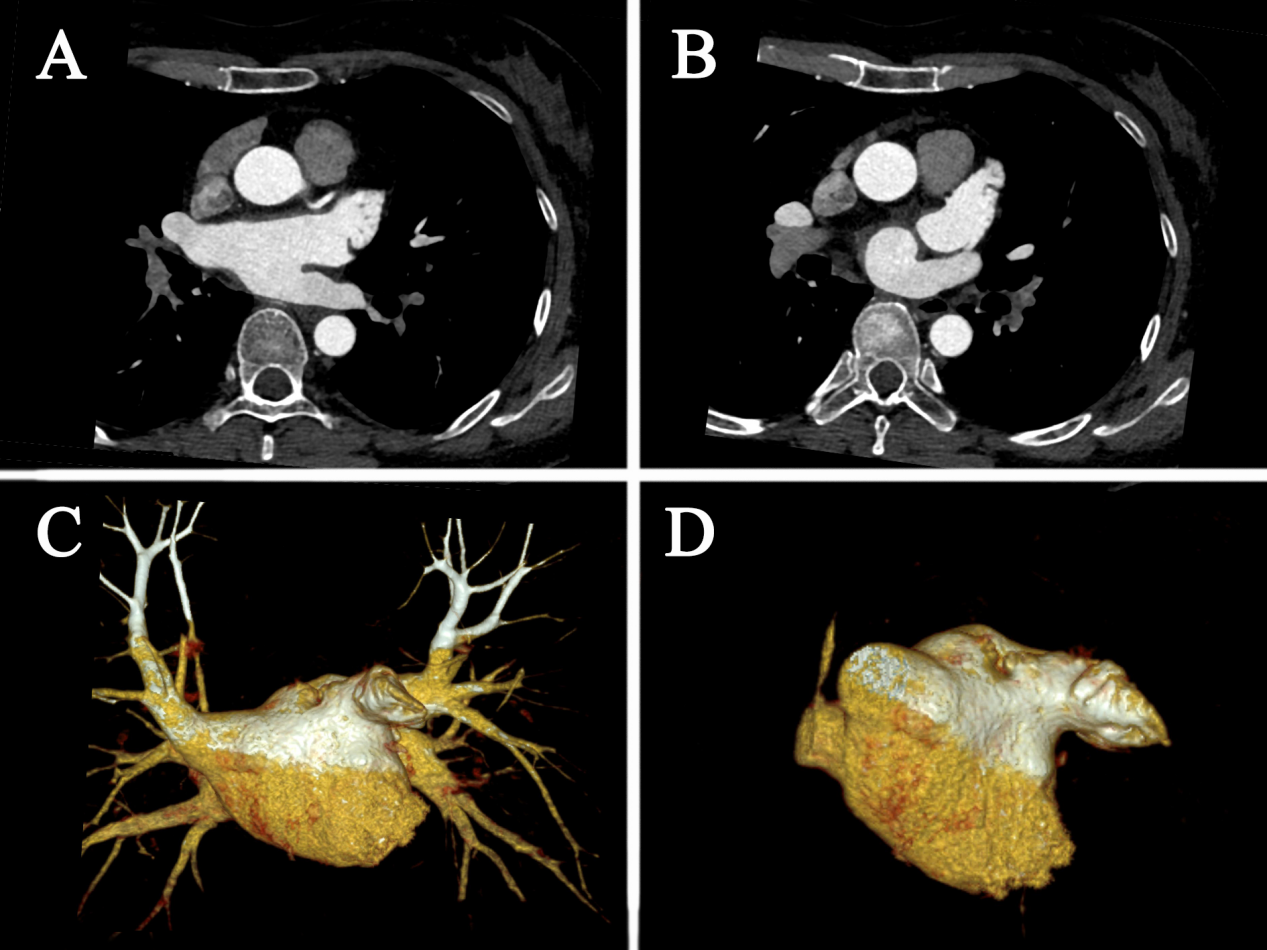
**Cardiac CT images**. (A) axis position; (B) axis position; (C) volume rendering; (D) volume rendering. CT, computed tomography.

Although LA diameter is a commonly used parameter in AF research, it has been well demonstrated that LA volume measured by CT or MRI is superior in accuracy and confidence of measurements and prognostic ability compared to LA volume measured through other examination methods [36]. Recent studies have suggested that not only a larger LA diameter [37, 38] but also greater LA wall thickness [39], sphericity [14, 40], box surface ratio [41], volume [42, 43], and volume index [44, 45, 46, 47] and lower ejection fraction [42, 45, 48] are indicative of late postoperative AF recurrence. This may be due to the adverse remodeling of LA, which leads to AF recurrence after CA. A growing body of evidence also shows that pulmonary veins (PVs)’ morphology and orientation correlate with AF recurrence [46, 49, 50]. Moreover, cardiac enhancement CT is a non-invasive and useful method for assessing the anatomy of the LA and PV and a more precise tool to estimate the left atrial appendage (LAA) [37, 38]. Previous studies have found that a larger LAA area, volume, and lower ejection fraction, as measured by CT preoperatively, marked poor surgical outcomes and correlated with late AF reappearance in patients undergoing CA [37, 42, 43, 48, 51, 52]. More particularly, the LAA contrast defect shown on cardiac enhancement CT images preoperatively was a non-invasive predictor of AF recurrence [53, 54]. This is probably because LAA CT contrast defects result from LAA function decline, which is associated with a greater incidence of recurrence after CA [53]. Therefore, CT examination before CA is expected to be a valuable predictor of AF recurrence after CA. Fig. 2 illustrates the cardiac CT images.

**Fig. 3.**
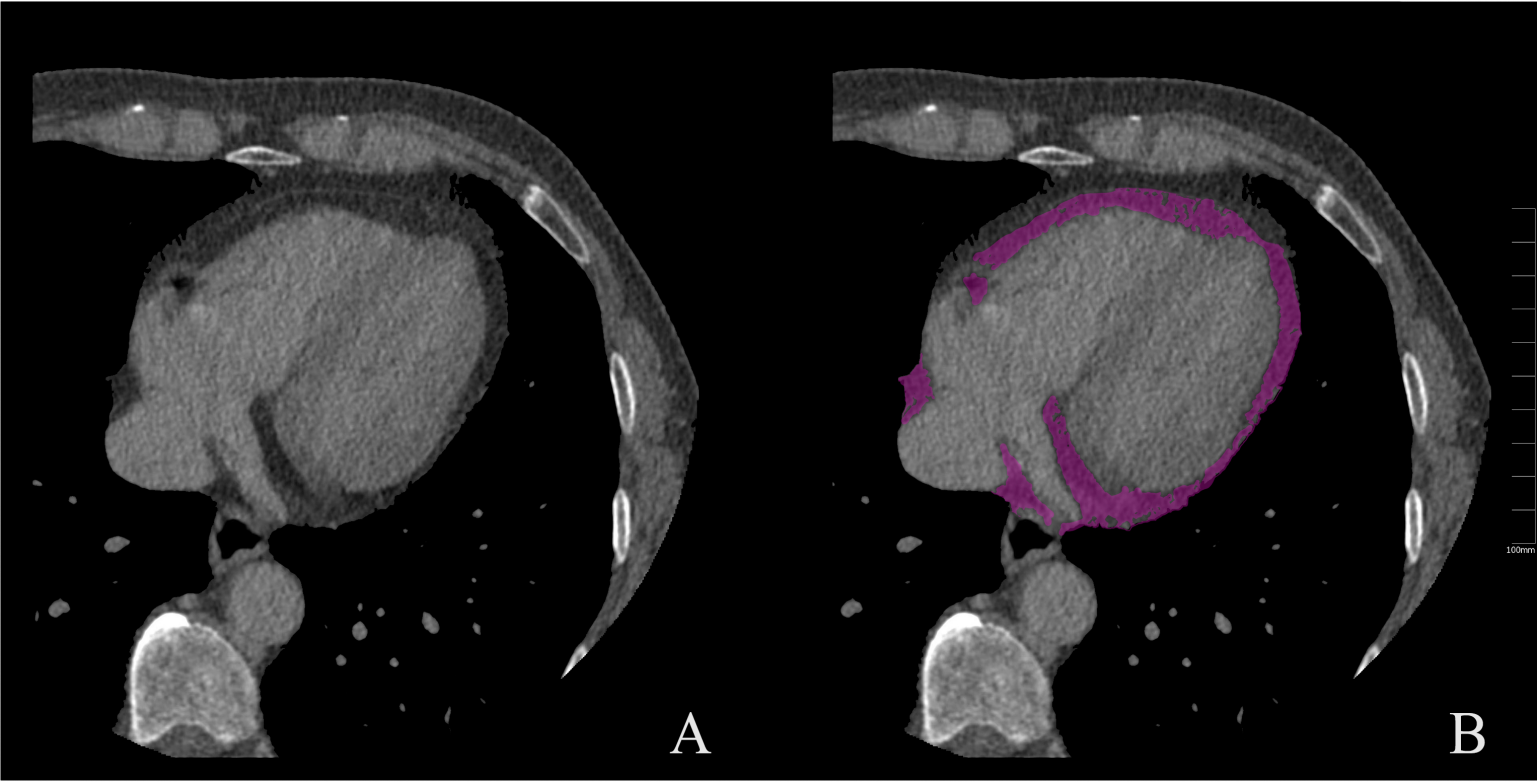
**Measurement of EAT on CT images**. (A) unenhanced CT image; (B) measurement of EAT (purple). CT, computed tomography; EAT, epicardial adipose tissue.

Given that it shows EAT more clearly than other imaging methods, CT is increasingly employed for EAT measurements [28, 55, 56]. Except for EAT volume, its attenuation, a novel indicator that has emerged in recent years, has been demonstrated to be an independent predictor of AF recurrence after CA [56, 57, 58]. Huber *et al*. [28] found that the EAT of the LA measured on cardiac enhancement CT images was independently associated with AF recurrence after CA. Recent meta-analyses have also reported that patients with AF recurrence after CA had larger EAT volumes than those who did not experience recurrence [55]. Ciuffo *et al*. [58] showed that the quality of peri-LA adipose tissue measured using CT attenuation was associated with recurrence after initial CA in patients with paroxysmal or persistent AF. Similarly, Beyer *et al*. [56] discovered that a higher EAT volume and lower attenuation observed on CT angiography before CA predicted AF recurrence after CA. The detection of AF recurrence by EAT was probably attributed to the sympathetic imbalance associated with AF recurrence, while cholinergic activity promoted inflammation and lipid accumulation in the EAT secretome and epicardial stromal microenvironment [59]. Few studies have also observed that interatrial septum fat volume [60], pericardial fat volume [61], and pericoronary adipose tissue attenuation [62] are linked to an increased risk of AF recurrence, which warrants further clinical investigation. Studies that used CT to predict recurrence following CA in patients with AF are summarized in Table 2 (Ref. [14, 28, 37, 38, 39, 40, 41, 42, 43, 44, 45, 46, 47, 48, 49, 50, 53, 54, 56, 58, 60, 61, 62]). Fig. 3 shows the measurement of EAT on CT images.

**Table 2.** **Prediction of AF Recurrence after Catheter Ablation by CT**.

Author (reference)	Sample size	Risk factors	Follow-up time	Predictive performance
Bisbal *et al*. [14]	243	LA sphericity	12 months	AUC = 0.687
Huber *et al*. [28]	212	LA enhancing EAT	12 months	-
Du *et al*. [37]	108	LAA volume >9.99 mL	12 months	AUC = 0.733
Kocyigit *et al*. [38]	359	LA diameter and cauliflower-type LAA morphology	37 months	-
Nakatani *et al*. [39]	213	Thick and heterogeneous LA wall	12 months	-
Moon *et al*. [40]	148	LA sphericity	12 months	AUC = 0.772
Keçe *et al*. [41]	70	Small box lesion surface area as a ratio of total LA surface area	13 (IQR: 10, 17) months	-
Tian *et al*. [42]	83	LAA ejection fraction and volume	19 (range 4–24) months	AUC = 0.817, 0.82
Straube *et al*. [43]	1103	LA volume and LAA volume	19 months	AUC = 0.63, 0.59
Strisciuglio *et al*. [44]	352	LA volume index	19 (IQR: 12, 24) months	-
Oka *et al*. [45]	292	LA ejection fraction, and indexed maximum and minimum LA volume	3 years	AUC = 0.666, 0.564, 0.611 (single procedure); 0.701, 0.616, 0.616 (multiple procedures)
Istratoaie *et al*. [46]	80	Large LA volume index and variant PV anatomy	14 (IQR: 12, 15) months	AUC = 0.713
Maier *et al*. [47]	415	LA volume index, BMI and type of AF	53 (IQR: 34.5, 73) months	AUC = 0.647
Kaufmann *et al*. [48]	50	Ejection fraction of LA and LAA	229 days	AUC = 0.94, 0.96
Mamchur *et al*. [49]	230	Large diameters and ovality of the left superior PV	14 (IQR: 12, 24) months	-
Li *et al*. [50]	97	Major diameter and cross-sectional area of right inferior pulmonary vein	12 months	AUC = 0.665, 0.659
				C-index = 0.766, 0.758
Nakamura *et al*. [53]	283	LAA CT contrast defect	858 days	-
Kawaji *et al*. [54]	1019	Severe filling defect in LAA in contrast CT	4.4 ± 2.0 years	-
Beyer *et al*. [56]	732	LA wall thickness, EAT volume and EAT attenuation	12 months	AUC = 0.649
Ciuffo *et al*. [58]	143	LA fat attenuation	12 months	-
Samanta *et al*. [60]	55	Interatrial septal fat volume, LA volume index, EAT volume and LA low-voltage area	11 ± 3 months	-
Goldenberg *et al*. [61]	130	EAT volume and pericardial fat volume	19.5 ± 8.5 months	-
Nogami *et al*. [62]	364	Pericoronary adipose tissue attenuation and EAT volume	26 (IQR: 19, 42) months	AUC = 0.726

CT, computed tomography; AF, atrial fibrillation; LA, left atrial; BMI, body mass index; EAT, epicardial adipose tissue; AUC, area under the curve; IQR, inter quartile range; LAA, left atrial appendage; PV, pulmonary vein.

## 4. Use of MRI for the Prediction of AF Recurrence after CA

Cardiovascular MRI can assess both LA structure and function in a single examination, thereby serving as an early indicator of AF recurrence [63, 64]. den Uijl *et al*. [64] demonstrated that LA volume, as determined by preoperative cardiovascular MRI, is a powerful predictor of AF recurrence after CA. Further studies have shown that, in addition to LA volume, RA volume and biatrial volume quantified by MRI can also predict AF recurrence after CA [65]. Alternatively, LA shape has been found to provide prognostic information for CA treatment [14, 66]. Bieging *et al*. [66] revealed that LA shapes, such as rounder, shorter, and more laterally rotated appendages, were associated with recurrence at postoperative follow-up of patients with AF undergoing CA. In a real-world study, LA sphericity measured using preoperative cardiovascular CT or MRI was the strongest predictor of AF ablation outcomes in patients with AF [14]. In contrast, Varela *et al*. [67] showed that LA vertical asymmetry is a new preoperative MRI index for differentiating patients with AF recurrence from those without. Moreover, an increasing number of studies have found impaired LA function parameters obtained by cardiovascular MRI, including decreased booster pump [63], reservoir strain [68], expansion index [68], emptying fraction [69], and ejection fraction [70], to be independently related to AF recurrence following CA therapy.

**Fig. 4.**
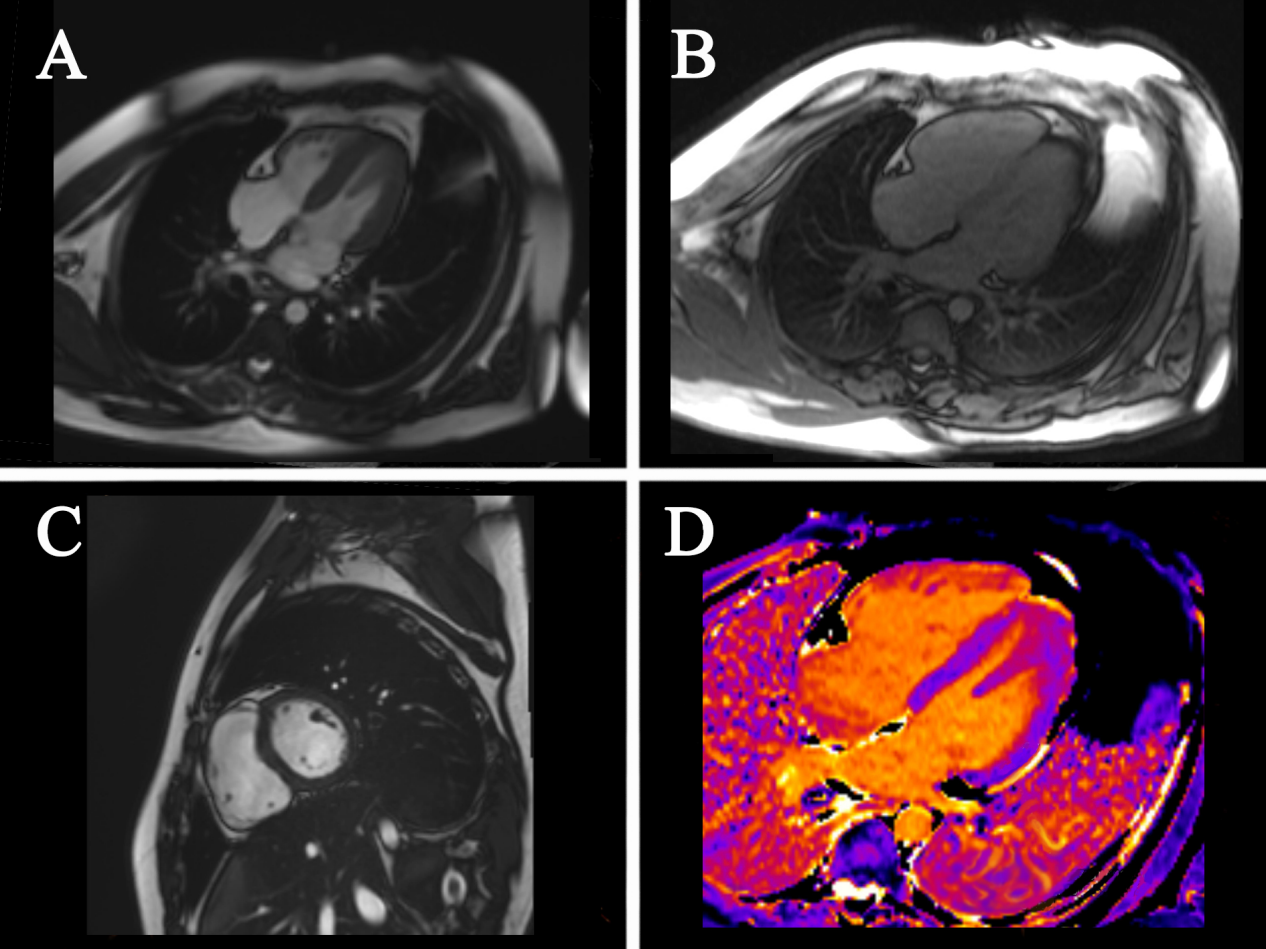
**Cardiac MRI images**. (A) four-chamber cardiac images; (B) four-chamber cardiac images; (C) cardiac short-axis images; (D) T1 mapping. MRI, magnetic resonance imaging.

LA fibrosis was also evaluated on MRI late gadolinium-enhanced images, which performed well in predicting the risk of recurrence following CA in patients with AF [13, 70, 71, 72]. Nevertheless, a recent study suggested no difference in LA and RA fibrosis between patients with and without AF recurrence [73]. Caixal *et al*. [74] further probed and found that the extent of LA fibrosis, especially near the descending aorta, was more predictive of the late recurrence of AF post-CA than was LA. Previously, Suksaranjit *et al*. [75] also reported that the higher the degree of structural remodeling of LAA in patients with AF with gadolinium enhancement quantification, the higher the risk of recurrence following CA. These findings may provide novel therapeutic targets in clinical settings. A controversial issue is whether LV fibrosis determined by MRI extracellular volume fraction measurements before CA is associated with AF recurrence [76, 77]. Gunasekaran *et al*. [76] discovered that LV extracellular volume fraction was irrelevant to AF recurrence following CA, whereas Li *et al*. [77] found that LV extracellular volume fraction predicted AF recurrence following CA. Further investigations are warranted to determine the importance of extracellular volume fraction in AF recurrence. Additionally, intra-atrial dyssynchrony during sinus rhythm [78] and total relative gap length in the ablation line [79] evaluated via MRI was also proven to be risk factors for AF recurrence in patients following CA. Accordingly, the preoperative MRI of patients with AF may be useful for the early identification of CA recurrence. A summary of studies that used MRI to predict recurrence after CA in patients with AF is presented in Table 3 (Ref. [13, 14, 63, 64, 65, 66, 67, 68, 69, 70, 72, 74, 75, 77, 78, 79]). Fig. 4 demonstrates the cardiac MRI images.

**Table 3.** **Prediction of AF Recurrence after Catheter Ablation by MRI**.

Author (reference)	Sample size	Risk factors	Follow-up time	Predictive performance
Chelu *et al*. [13]	308	LA fibrosis	5 years	-
Bisbal *et al*. [14]	243	LA sphericity	12 months	AUC = 0.687
Gastl *et al*. [63]	52	Impaired LA booster pump function	12 months	AUC = 0.73
den Uijl *et al*. [64]	83	Large LA volume	12 months	AUC = 0.680
Kumagai *et al*. [65]	100	Large LA volume, RA volume and biatrial volume	8 months	AUC = 0.73, 0.78 and 0.79
Bieging *et al*. [66]	254	More round LA shape with a shorter, more laterally rotated appendage	475 days	C-statistic = 0.72
Varela *et al*. [67]	144	LA sphericity, LA vertical asymmetry	12, 24 months	AUC = 0.68, 0.65 (12 months); 0.63, 0.63 (24 months)
Benjamin *et al*. [68]	80	Large minimum LA volume, and low LA reservoir strain and expansion index	12 months	-
Habibi *et al*. [69]	51	Low LA emptying fraction	12 months	-
Chubb *et al*. [70]	89	Low LA and LV ejection fraction, and LA fibrosis	24 months	AUC = 0.646, 0.639 and 0.661 (150 days postprocedure)
Csécs *et al*. [72]	55	LA fibrosis	12 months	AUC = 0.768
Caixal *et al*. [74]	108	LA fibrosis especially near the descending aorta	12 months	AUC = 0.65
Suksaranjit *et al*. [75]	74	LAA structural remodeling	18 months	-
Li *et al*. [77]	130	LV extracellular volume fraction expansion	13 (IQR: 12, 16) months	-
Ciuffo *et al*. [78]	208	Intra-atrial dyssynchrony during sinus rhythm	20 ± 6 months	C-statistics = 0.77
Linhart *et al*. [79]	94	Total relative gap length in the ablation line	15 ± 10 months	-

MRI, magnetic resonance imaging; AF, atrial fibrillation; LA, left atrial; RA, right atrial; LV, Left ventricle; AUC, area under the curve; LAA, left atrial appendage; IQR, inter quartile range.

## 5. Use of ML for the Prediction of AF Recurrence after CA

ML based on medical imaging is not limited by the maximum limit of grayscale that the human eye can observe, thus observing lesion features that cannot be recognized by the naked eye and providing more information for disease assessment [80, 81]. ML is widely used in the diagnosis, differential diagnosis, and prognosis evaluation of various diseases and plays an increasingly important role in assessing AF recurrence [82, 83, 84]. As mentioned above, EAT is closely related to AF recurrence [27, 28]. It has been demonstrated that the assessment of EAT based on CT radiomics is a more promising tool for predicting AF recurrence than EAT volume and attenuation [85]. Yang *et al*. [86] discovered that the radiomic signatures of EAT based on CT image extraction were associated with recurrence within 1 year after CA in patients with AF and that the area under the curve values of their constructed prediction models were 0.808 and 0.793, respectively. In addition, Ilyushenkova *et al*. [85] found that EAT-based normalized gray-level nonuniformity strongly predicted late postoperative CA in patients with lone AF compared to other risk factors.

Furthermore, ML research has increasingly been applied to identify AF recurrence by LA and PVs in CT images [83, 84]. Chen *et al*. [84] constructed a model to measure LA volume using a deep learning approach, which allowed the prediction of AF recurrence 1 and 2 years after CA. Atta-Fosu *et al*. [87] used CT angiography and applied ML algorithms to identify LA shape features, showing that shape differences in the area around the LA and PVs were associated with the late recurrence of AF. Furthermore, Labarbera *et al*. [88] and Firouznia *et al*. [83] reported that not only LA morphology but also alterations in PVs could be novel imaging markers for patients with AF recurrence after CA; therefore, they used ML to extract the radiomic features of LA and PVs, respectively, and demonstrated that they could be good predictors for AF recurrence. It is worth noting that MRI-based ML will strengthen the identification of LA fibrosis by late gadolinium-enhanced MRI, thus improving the predictive ability for AF recurrence [89, 90]. Moreover, deep learning-based LA-curved M-mode speckle tracking also provides a novel method to predict AF recurrence following CA [91]. Therefore, following extensive studies, ML in non-invasive imaging is expected to be a superior imaging biomarker in the future, improving the early detection and prevention of recurrence in patients with AF undergoing imaging evaluation. The findings of studies using ML to predict recurrence following CA in patients with AF are summarized in Table 4 (Ref. [83, 84, 85, 86, 87, 88, 89, 91]).

**Table 4.** **Prediction of AF Recurrence after Catheter Ablation by ML**.

Author (reference)	Sample size	Methods	Risk factors	Follow-up time	Predictive performance
Firouznia *et al*. [83]	203	CT-ML	Morphological and texture variation of the LA and PV	-	AUC = 0.70, 0.78
Chen *et al*. [84]	58	CT-deep convolutional neural networks	LA volume and LA volumes normalized by the body surface area	12 and 24 months	AUC = 0.742, 0.736; 0.696, 0.684
Ilyushenkova *et al*. [85]	63	CT-rediomics	EAT-gray level nonuniformity normalized	12 months	AUC = 0.809
Yang *et al*. [86]	314	CT-ML	LA-EAT radiomic signatures and volume	12 months	AUC = 0.915, 0.808 (training); 0.853, 0.793 (validation)
Atta-Fosu *et al*. [87]	68	CT-ML	Shape features of the LA and areas around the PV	12 months	AUC = 0.67
Labarbera *et al*. [88]	150	CT-rediomics	Greate right carina angle, reduced anterior-posterior atrial diameter, greater atrial volume normalized to height, and steeper right inferior pulmonary vein angle.	12 months	AUC = 0.68
Shade *et al*. [89]	32	MRI-ML	LA	366 (IQR: 365, 467) days	AUC = 0.82
Hwang *et al*. [91]	606	Curved M-mode speckle-tracking images-deep convolutional neural network	LA	-	AUC >0.8

ML, machine learning; CT, computed tomography; MRI, magnetic resonance imaging; AF, atrial fibrillation; LA, left atrial; PV, pulmonary vein; AUC, area under the curve; EAT, epicardial adipose tissue; IQR, inter quartile range.

## 6. Discussion

Preoperative imaging for the non-invasive prediction of AF recurrence following CA has demonstrated encouraging results, as the value of imaging for the assessment of structural and functional changes in the hearts of patients with AF is superior to that of clinical and laboratory tests. Moreover, imaging can provide incremental value in predicting recurrence following CA in patients with AF through the evaluation of LA fibrosis and EAT. However, there are some limitations to assessing the efficacy of CA in patients with AF using imaging modalities. First, although studies have found that larger LA diameter and volume and lower ejection fraction are independent risk factors for AF recurrence after CA, there is a lack of consensus on this “greater and lesser” phenomenon owing to individual and racial differences [16, 21]. Furthermore, the anatomy of the LA and PVs is complicated, rendering manual measurement and profiling more time-consuming [87]. There is also often interobserver variability in the morphological assessment of LA, LAA, and PVs, which would lead to different predictive outcomes pertaining to recurrence following CA. Second, while LA fibrosis estimated on MRI gadolinium-enhanced images shows promising performance in predicting the risk of recurrence after CA in patients with AF, it should only be performed in patients for whom MRI gadolinium-enhanced images of sufficient quality can be acquired to quantify fibrosis. Furthermore, compared with conventional LV-MRI gadolinium enhancement, LA-MRI gadolinium enhancement requires high spatial resolution, patient-specific scan parameters, and strict contrast dosing [13]. Finally, evaluating EAT opens a novel window for the non-invasive prediction of recurrence after CA in patients with AF; however, the manual segmentation of EAT is time-consuming and challenging. The normal range of reference values for EAT in clinical settings remains unknown.

Future studies with larger sample sizes and more centers are needed to determine a range of reference values for LA and EAT. Moreover, appropriate LA-MRI gadolinium enhancement scan parameters should be established through technical exploration. With the development of artificial intelligence, ML based on medical images can not only discover imaging features that cannot be recognized by the naked eye but also fully realize the automatic segmentation of LA, PVs, and EAT quickly and effectively, immensely improving the efficiency and accuracy of the physician’s assessment, and therefore showing potential for clinical application as a non-invasive method for the prediction of AF recurrence following CA. However, current studies on medical imaging-based ML for AF recurrence are scarce and have small sample sizes; thus, further research is warranted.

Furthermore, preoperative imaging for safety and efficacy assessment of AF ablation is a direction worth exploring but remains controversial. Di Cori *et al*. [92] showed that preprocedural CT does not improve the safety and efficacy of AF ablation but rather significantly increases the cumulative radiological exposure. Teres *et al*. [93] found that personalized AF ablation with a customized ablation index based on CT-measured LA wall thickness was feasible, effective, and safe. Marrouche *et al*. [94] demonstrated that using MRI-guided fibrosis ablation for persistent AF did not significantly reduce the recurrence rate.

## 7. Conclusions

In summary, echocardiography, CT, and MRI are meaningful in predicting recurrence after CA in patients with AF and can assist cardiologists in clinical decision-making and improve the long-term prognosis of patients with AF. Medical imaging-based ML may have the potential for clinical application, but large, multicenter, prospective studies are needed to confirm its value in assessing AF recurrence. With ongoing progress in imaging technology and artificial intelligence, imaging is expected to accurately identify patient groups at a higher risk of recurrent AF preoperatively, thus avoiding unnecessary risks associated with non-invasive surgery.

## Abbreviations

AF, atrial fibrillation; CA, catheter ablation; CT, computed tomography; MRI, magnetic resonance imaging; ML, machine learning; LA, left atrial; LV, left ventricular; E/e’, the ratio of mitral inflow velocity to mitral annular tissue velocity; EAT, epicardial adipose tissue; STE, speckle tracking echocardiography; 2D, two-dimensional; RA, right atrial; PVs, pulmonary veins; LAA, left atrial appendage.
